# Cation-Induced Pesticide Binding and Release by a Functionalized Calix[4]arene Molecular Host

**DOI:** 10.1038/srep08982

**Published:** 2015-03-11

**Authors:** Li Luo, Xiaoyan Zhang, Ningmei Feng, Demei Tian, Hongtao Deng, Haibing Li

**Affiliations:** 1 Key Laboratory of Pesticide and Chemical Biology (CCNU), Ministry of Education, College of Chemistry, Central China Normal University, Wuhan 430079 (P. R. China)

## Abstract

Ion-controlled switchable progress is very important in many biological behaviors. Here, we reported K^+^-controlled switch, this switch system exhibited excellent carbaryl (G) binding/release by fluorescent (FL), ultraviolet-visible (UV) spectrums and ^1^H NMR spectroscopy. More importantly, the K^+^-controlled G binding/release switch based on C4C5 not only in the solution, but also on the surface, promising for the application for the pesticide controlled release.

Ion-controlled switchable process is very important in many biological behaviors, for example, metal ion-directed protein folding and self-assembly, Ca^2+^ induced contraction or relaxation of the human heart and the Na^+^ stimulated nerve impulses^1^,^2^,^3^,^4^,^5^,^6^. Recently, those in which the binding substrate could be “on” or “off” by ion, have attracted great interest in host-guest complex^7^,^8^,^9^, due to their extensive potential application not only in the construction of artificial molecular machines but also in the development of sensing and controllable drug-delivery system^10^,^11^,^12^,^13^,^14^,^15^,^16^,^17^,^18^,^19^,^20^,^21^,^22^,^23^. For example, riptycene-derived containing two dibenzo-[18]-crown-6 interacted with paraquat derivatives which can be combined and released by Li^+^^24^. To our knowledge, most biological ion-controlled switches realize on the membrane interface, but the ion-controlled switch on the surface has been scarcely reported.

Controlling surface wettability has attracted interest in recent years^25^,^26^,^27^,^28^, as it is important for range of biological and chemical application^29^,^30^, thus, chemical, thermal, and pH-sensitive wettability changed have been reported. These changes own to their inherent physicochemical properties under environmental conditions. These means can provide a general method to realize ion-controlled switch between hydrophobicity and hydrophilicity. For example, wettability provides a method for calix[4]azacrown modified Si-surface response to [C4min]Cl^31^. Among of these, the design and synthesis of response molecules play an important role in constructing wettable functional surface. As we all known, calixarene, because of its adjustable cavity and modifying substituents of the upper and lower rims, have been a good choice of functional response molecules. Calixcrown make a family of calixarenes that exhibit a superior recognition of organic molecules and metal ions via the cooperation of both the calixarene and crown moieties^32^,^33^,^34^,^35^,^36^,^37^,^38^,^39^. Carbaryl (**G**) is a pesticide which have naphthalene group can interact with calixarene through π-π stacking. Crown ether was employed to selectively bind K^+^ in aqueous solution^40^. It was known that **18C6** is a very strong sequestering agent for potassium ion^41^,^42^, which has strong binding affinity toward K^+^. Therefore, we designed a wettable responsive switch based on K^+^-controlled calixcrown binding/release **G**. All were shown in the figure 1b. As a consequence of its outstanding properties, the K^+^-controlled switch has formed attractive application in many fields, especially on a silicon surface.

## Results

### Synthesize of C4C5

**Figure 1:**
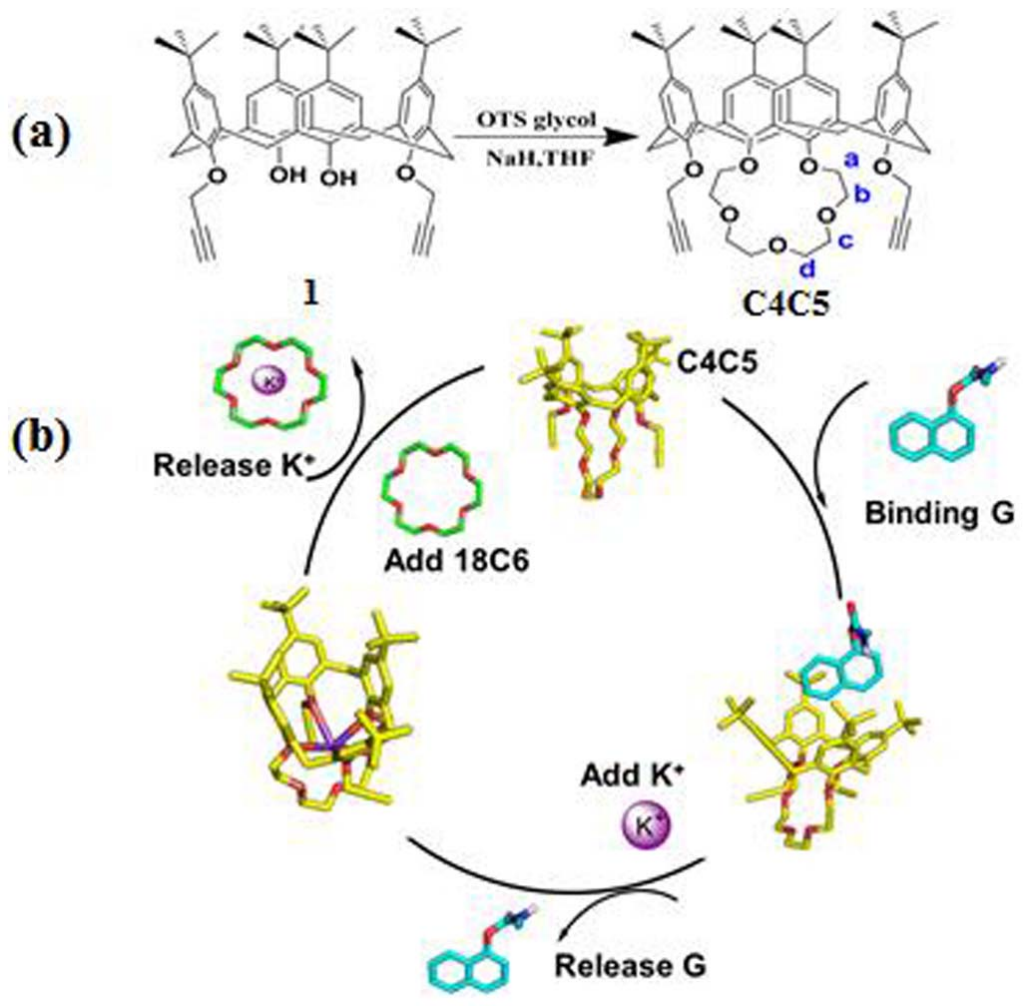
(a) The synthesis of **C4C5**. (b) The scheme of K^+^-controlled binding/release **G**.

Dialkyne **1**^43^,^44^,^45^ (0.5 g, 9.8 mmol) and NaH (0.067 g, 40 mmol) and OTS glycol (0.35 g, 10 mmol) were stirred in the anhydrous THF (70 mL), see in the figure 1a. The mixture reaction was stirred at 50°C for 15 h. When the flask was cooled to room temperature, methanol was added to remove the no reacting NaH. The residue solvent was evaporated by suction filtration to give white powder **C4C5** (0.45 g, 73%). The structure and conformation of **C4C5** were confirmed by ^1^H NMR, ^13^C NMR studies (Supplementary Fig. S1, Fig. S2), elemental analyses and EI(+)MS spectra (Supplementary Fig. S3). All of these indicated the cone conformation of **C4C5**^46^,^47^,^48^,^49^. Base on the interaction between the cavity of the **C4C5** and naphthalene group, we obtained [**C4C5**+**G**] complex with a conjugation ratio of 1:1, by UV, ^1^H NMR spectroscopy and density functional calculation studies. Further, in the situ [**C4C5**+**G**] complex was added K^+^. From fluorescence and ^1^H NMR spectroscopy we found the **C4C5** binding/release **G** was controlled by K^+^. Even further, the K^+^-controlled **G** binding/release switch had been successfully applied on the functional micro-silicon surface by the simple click reaction. Moreover, the K^+^-controlled **G** binding/release could act as a convenient and effective wettability switch by contact angle (CA) measurement on the silicon surface.

### The K^+^-controlled G binding/release in the solution

**Figure 2:**
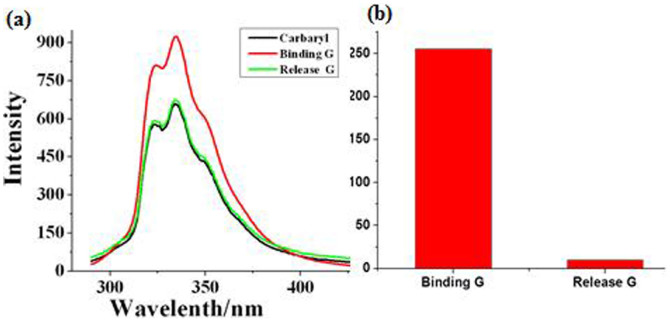
(a) Fluorescence intensity changes of **G**, binding **G** and release **G** in CH_3_CN upon irradiation with 334 nm light (λex = 280 nm, black line represents the fluorescence of **G**; red line represents binding **G**; green line represents release **G**). (b) FL variation [ΔI = (I-I_0_)] (I_0_ is the FL intensity of **G**) histogram for the [**C4C5**+**G**] complex and [**C4C5**+**G**] with K^+^ at 334 nm.

To provide support for the K^+^-controlled binding/release **G**, some experiments were done in the solution phase. First of all, a fluorescence experiment of the **C4C5** and **G**, K^+^ were carried out using an excitation wavelength of 280 nm. The fluorescence of **G** (1.0 × 10^−5^ M, 2.0 mL) enhanced with the addition of the **C4C5** (1.0 × 10^−3^ M, 20 μL) in CH_3_CN because of their interaction. This indicated the [**C4C5**+**G**] complex was successfully obtained. To further study of the **C4C5** with **G** carried out by K^+^, the same equiv. of K^+^ was added for the interaction with [**C4C5+G**] which showed fluorescence recovered (Fig. 2). Then, ultraviolet-visible (UV) spectrums of **C4C5**, [**C4C5**+**G**], [**C4C5**+K^+^] were also done in CH_3_CN solution. From the spectrum of UV (Supplementary Fig. S4), the absorption of **C4C5** was increased when **G** added and recovered when K^+^ added. The fluorescence spectrum and UV spectrum all demonstrated K^+^- controlled binding/release **G** can come true in the solution.

In order to illustrate the details of formation of [**C4C5**+**G**] in the solution, the binding stoichiometry of the complex formed between **C4C5** and **G** was 1:1 from Job's plot by ultraviolet-visible (UV) spectrum, which had a peak at 277.9 nm with a molar fraction of 0.5 (Supplementary Fig. S5). The binding stoichiometry of the complex formed between **C4C5** and K^+^ was also 1:1 from the Job's plot. The liner relationship between intensity of UV at 277.9 nm and different concentration of **G** was carried (Supplementary Fig. S6). We found the [**C4C5**+K^+^] binding stoichiometry was also 1:1 (Supplementary Fig. S6). The host **C4C5** was 1 × 10^−4^ M, 2.0 mL CH_3_CN solution and then the different amount of **G** (1 × 10^−3^ M, CH_3_CN solution) was gradually adding into the host solution. At the same time, the liner relationship between intensity of UV 277.9 nm and different concentration of K^+^ was also carried using the same method (Supplementary Fig. S7). The association constant of **C4C5** and **G** is 6.22 × 10^3^ M^−1^, however, **C4C5** and K^+^ is 1.04 × 10^5^ M^−1^, these results clearly suggested the complex of [**C4C5**+K^+^] was much stable than the complex of [**C4C5**+**G**].

### The K^+^-controlled G binding/release characterized by NMR

**Figure 3:**
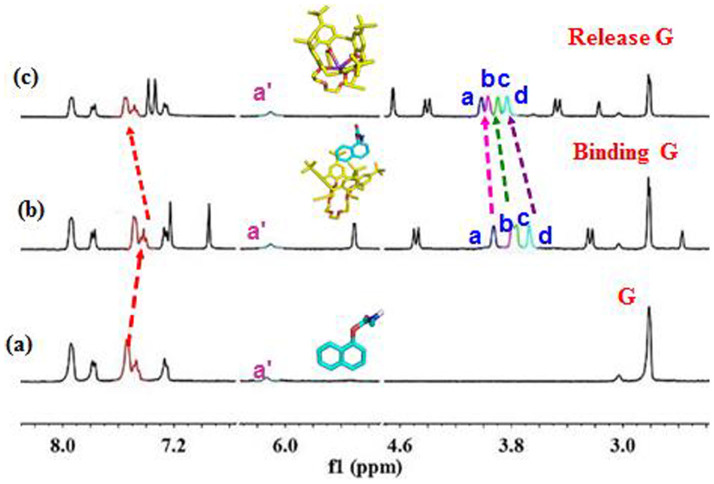
(a) Partial ^1^H NMR spectroscopy of **G** (6.0 mM each, CD_3_CN, 400 MHz, 298 K). (b)Partial ^1^H NMR spectroscopy of **C4C5** reacted with **G**. (c) ^1^H NMR spectroscopy of K^+^ and **C4C5**, which indicated **G** release from the **C4C5** by K^+^ interacted with crown ring with **C4C5**.

To obtain insights into the mechanism, NMR experiments were carried out, 6.0 mM **C4C5** and 1.0 equiv. of **G** and K^+^ in CD_3_CN, as showed in Fig. 3. The protons of the napthalene in the **G** underwent upfield shift of 0.05 ppm in the presence of **C4C5**. The result also indicated that the [**C4C5**+**G**] complex was successfully formed. When K^+^ added, the protons of naphthalene underwent an upfield shift of 0.05 ppm and recovered to original chemical shift, moreover, the protons of crown have underwent the protons of crown ether unit of **C4C5** H_a_, H_b_, H_c_ and H_d_ underwent downfield shift of 0.04 ppm, 0.18 ppm, 0.14 ppm and 0.17 ppm respectively. According to the NMR, we deduced that before K^+^ was added, the crown ring of **C4C5** was stretched, when K^+^ added, K^+^ entered into crown space, which made the lower space of crown become smaller and this reaction has made the space of the upper of the **C4C5** become bigger and led to **G** released.

We also found the **18C6** was added (Supplementary Fig. S8e), the chemical shifts of H_a_, H_b_, H_c_, H_d_ recovered to original values, moreover there appeared a new in peak in the spectrum, we supposed the new peak is the complex of **18C6** and K^+^. Based on above experiments, we have obtained the K^+^-controlled **G** binding/release switch.

### The K^+^-controlled G binding/release characterized by ESI-MS

**Figure 4:**
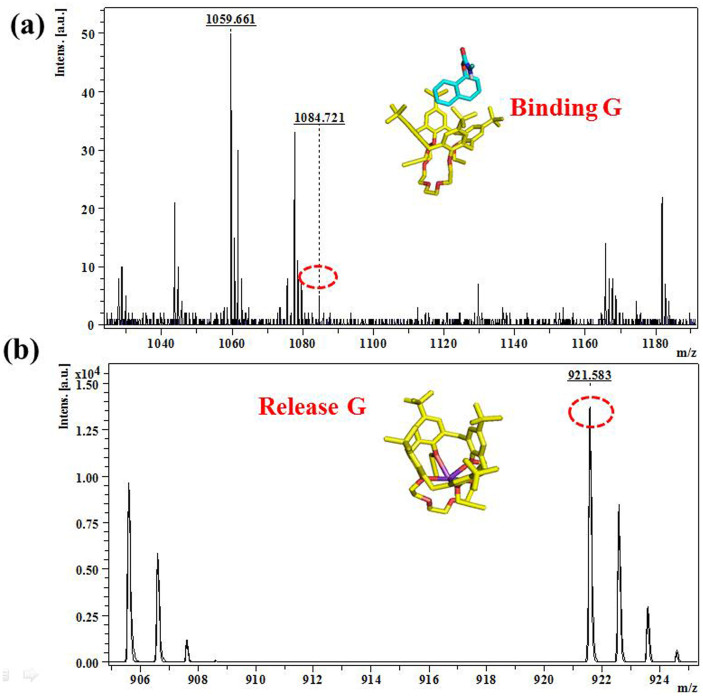
(a) **G** and **C4C5** were mixed, there appeared m/z = 1084.721 in the spectrum, which indicated [**C4C5**+**G**] has formed. (b) [**C4C5**+**G**] was added into KClO_4_, there has appeared m/z = 921.611 in the spectrum and m/z = 1084.72 was disappeared, which indicated **G** was released from **C4C5** and [**C4C5**+K^+^] was formed.

To further verify the binding between **C4C5** and **G**, the ESI-MS analysis was carried out. The peak observed at m/z = 1084.72 in the ESI mass spectrum (Fig. 4a) can be attributed to [**C4C5+G**] complex. Furthermore, the peak observed at m/z = 921.61 in the ESI mass spectrum (Fig. 4b) can be attributed to [**C4C5**+K^+^] complex, we also found m/z = 883.58 in the ESI mass spectrum (Fig. S9) can be attributed to K^+^ released from **C4C5**. The whole ESI-MS study fully explained the binding/release process and also confirmed the 1:1 complex formation **C4C5** and **G**, **C4C5** and K^+^.

### The Gaussian calculation of C4C5, [C4C5+G] and [C4C5 and K^+^]

**Figure 5:**
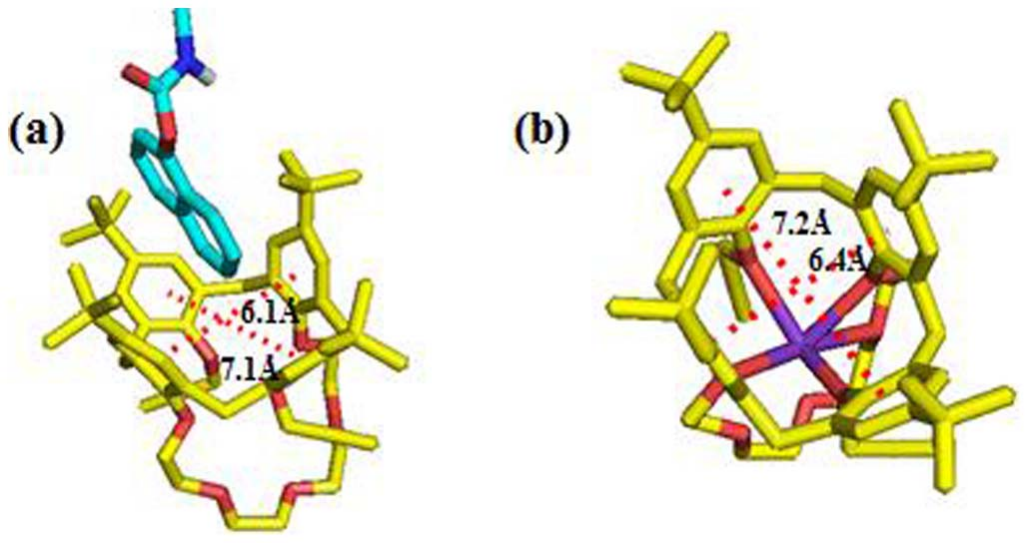
(a) The side view on the optimized structure of [**C4C5**+**G**] complex, the binding energy was −0.0014 a.u./mol (in the **C4C5** the yellow atoms represent carbon, red atoms represent oxygen, blue atoms represent nitrogen, white atom represents hydrogen; in the **G**, the cyan atoms represent carbon, the blue atoms represent nitrogen, red atoms represent oxygen). (b) The top view on the optimized structure of [**C4C5**+K^+^], the binding energy was −0.9438 a.u./mol (the yellow atoms represent carbon, red atoms represent oxygen, purple atom represents potassium). Which demonstrated the complex of [**C4C5**+K^+^] was much stable than [**C4C5**+**G**]. The space of [**C4C5**+K^+^] is 6.4Å, 7.2Å, which is much bigger than [**C4C5**+**G**] and make **G** release from the space.

Further, the binding of **C4C5** and **G**, K^+^ were also examined by Gaussian03. The results were shown in Fig. 5. The host **C4C5** was yellow, the guest carbaryl was green, oxygen atoms were red, nitrogen atoms were blue, hydrogen atoms were white, purple atom was potassium. The structure of **C4C5** and the complex of [**C4C5**+**G**], [**C4C5**+K^+^] have been optimized by going through a cascade process starting from HF/3-21G → HF/6-31G → B_3_LYP/3-21G → B_3_LYP/6-31G. Meanwhile, the frequency analysis calculations were performed and the absence of imaginary frequencies indicated the low energy minimum of the structures obtained. All the energy were obtained by the equation of ΔE (binding energy) = E (host − guest) − [(E (host) + E (guest)], [**C4C5+G**] binding energy is −0.0014 a.u./mol, [**C4C5+G+K^+^**] binding energy is 0.05789 a.u./mol, while [**C4C5**+K^+^] is −0.9438 a.u./mol, comparing to these energy, which indicated that [**C4C5**+K^+^] complex is much stable than [**C4C5**+**G**] complex. The space of [**C4C5**+**G**] is 6.1Å, 7.1Å, while [**C4C5**+K^+^] is 6.4Å, 7.2Å, which indicated that K^+^ interacted with **C4C5** made the space of become bigger and **G** can released from the space. These results of molecular mechanics calculation were generally consistent with the UV, ^1^H NMR experimental results. All the calculation details were shown in Supplementary Fig. S10, Fig. S11, Fig. S12.

### K^+^- controlled G binding/release on the surface

**Figure 6:**
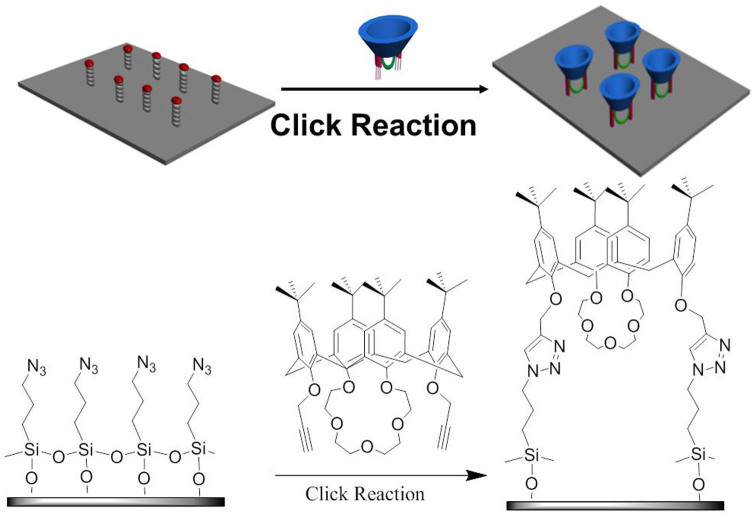
The formation process of the functional C4C5-SAM, which indicated that the C4C5 has successfully modified on the silicon surface.

More importantly, the K^+^-controlled binding/release carbaryl switch had an important and potential application by measurement of the contact angle (CA) on a functional micro- silicon surface. Because micro-structured and functional silicon surface can amplify the signal output with the respect of wettability^50^, it could reversibly switch between hydrophobic character and a hydrophilic character. At first, the **C4C5**-SAMs (self-assembled monolayer) were established by click reaction between the Si-N_3_ SAMs and **C4C5** in CH_3_CN (Fig. 6).

**Figure 7:**
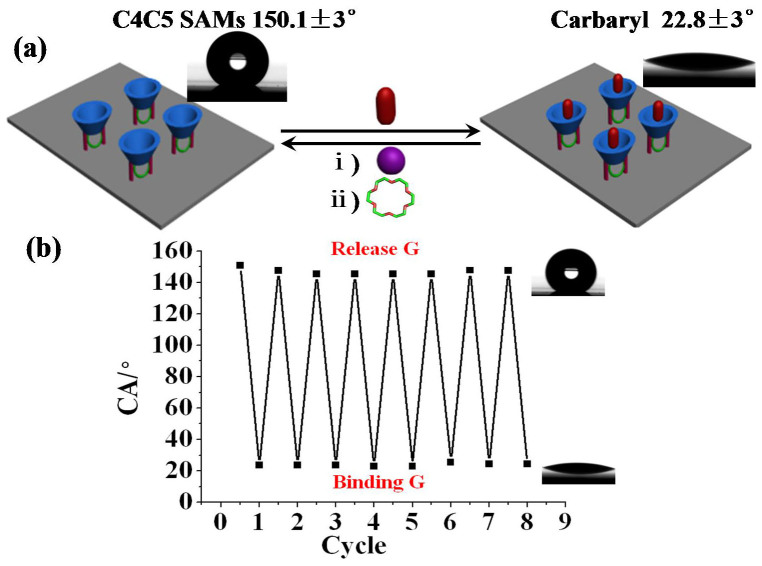
(a) CA relationship images for C4C5 SAMs with G, K^+^, 18C6. (b) Cycling experiment of K^+^-ion controlled switch which indicate a good reversibility between hydrophobicity and hydrophilicity.

Through the SEM (Supplementary Fig. S13), the rough substrate exhibited a regular array of square silicon micro convexes (bright squares), and then the **C4C5**-modified substrate showed a thin film on the surface. Comparing SEM image of the rough surface before and after modification with **C4C5**, these results indicated that **C4C5** was successfully modified on the silicon surface. From XPS (Supplementary Fig. S14), the concentration of carbon had a significant increase and the concentration of oxygen had an obvious decrease. Moreover, the water-drop profiles showed a different CAs on bare silicon surface. This also proved the above conclusion. The silicon surface was immersed into **G** solution (1.0 × 10^−3^ M), after 15 min the silicon wafer was washed by little water to remove the residue **G**, dried by nitrogen and measured. The results showed in Figure 7a, **C4C5** SAMs immersed into **G** the CA became from superhydrophobic to hydrophilic, which was only 22.8 ± 3.0°, however, when immersed into KClO_3_ solution (1.0 × 10^−3^ M) and **18C6** solution (1.0 × 10^−3^ M) respectively, the wettability of the surface recovered to their original values (150.0 ± 3.0°). Because **G** interacted with **C4C5** and the group of CONH was exposed, which made the surface become hydrophilicity, nevertheless, K^+^ formed complex with **C4C5** and made **G** released from the space and left the tertiary butyl, which led to the surface become superhydrophility again. **18C6** interacting with K^+^ didn't change the property of the upper rim of **C4C5**, so the wettability remained superhydrophobicity (Supplementary Fig. S15).

Upon further treatment of the surface with water, then the silicon was immersed into **G**, K^+^ and **18C6** consecutively, the results remained the same as the first time. A cycle experiment (Fig. 7b) reflected a good reversibility for K^+^-controlled **G** binding/release switch on the surface. According to above results, we found the experiments were consistent with NMR and Gaussian calculation, which confirmed K^+^-controlled and binding/release **G** based on the surface wettability can be established by contact angle measurement.

## Discussion

In conclusion, we found that the fluorescent intensity of **C4C5** and **G** increasing/decreased by K^+^. More interestingly, the application of wettability can provide a general method to realize K^+^-controlled switch between hydrophobicity and hydrophilicity. It is anticipated that this research can be further extended to fabricate functional surfaces for pesticide control release, intelligent microfluidic and laboratory-on-chip devices

## Methods

### Materials and instruments

^1^H NMR and ^13^C NMR were recorded on Varian Mercury VX400 instrument at ambient temperature with TMS as the internal standard. ESI-MS were recorded on a Finnigan LCQ-Advantage instrument. The static water contact angle was measured at 25°C by means of an OCA 20 contact angle system (Dataphysics, Germany). X-ray photoelectron spectroscopic (XPS) image was gained by PHI Quantera SXM. The scanning electron microscope image was measured at 25°C by means of a JSM-6700F HR-FESEM.

All chemicals were A.R. grade and were purified by standard procedures. Mill-Q water was used to prepare all solutions in this study.

### The synthesis of C4C5

Compound **1** (0.5 g, 9.8 mmol) and NaH (0.067 g, 40 mmol) and OTS glycol (0.35 g, 10 mmol) were stirred in the anhydrous THF (70 mL). The mixture reaction was stirred at 50°C for 15 h. When the flask was cooled to room temperature, methanol was added to remove the no reacting NaH. The residue solvent was evaporated by suction filtration to give white powder **C4C5** (0.45 g, 73%). ^1^H NMR (400 MHz, CH_3_CN): δ 7.22 (4H, s, Ar*H*), 6.95 (4H, s, Ar*H*), 5.50 (4H, s, C*H_2_*CCH), 4.48 (4H, d, *J* = 11.5 Hz, ArC*H_2_*Ar), 3.97 (4H, s, OC*H_2_*), 3.78 (8H, d, *J* = 10.7 Hz, OC*H_2_*C*H_2_*O), 3.68 (4H, s, OCH_2_C*H_2_*O), 3.23 (4H, d, *J* = 12.3 Hz, ArC*H_2_*Ar), 2.57 (2H, s, CC*H*), 1.27 (18H, s, C(C*H_3_*)_3_), 1.04 (18H, s, C(C*H_3_*)_3_). ESI(+)MS calcd for C_58_H_74_O_7_Na [M+Na]^+^ 905.5 found: 906.0; calc. for C_58_H_74_O_7_Na [M+Na+H]^+^ 906.5 found: 907. Anal. Calc. for C_58_H_74_O_7_: C, 78.74; H, 8. 38. Found: C, 78.66; H, 8.26%.

### Fluorescence experiments

All experiments of fluorescence were carried out at an excitation wavelength of 288.0 nm using a fluorescence spectrometer and a 1 cm quartz cell. **C4C5** solution (1.0 × 10^−5^ M) was made by dissolving **C4C5** in CH_3_CN (5.0 mL). **G** solution (1.0 × 10^−3^ M) was made by dissolving **G** in CH_3_CN (5.0 mL) The K^+^ solution was made at 1.0 × 10^−3^ M in CH_3_CN. The fluorescence was carried at 288.0 nm after adding the appropriate volume (20 μL) of C4C5 solution to measure the fluorescence. Then the K^+^ solution (20 μL) was added to the above mixed solution to measure the fluorescence, which showed a fluorescence recovery after adding K^+^.

### Ultraviolet-visible experiments

All experiments of UV were carried out using a UV spectrometer and a 1 cm quart cell. 1.0 mL1.0 × 10^−4^ **C4C5** interacted with **G** (100 μL, 1.0 × 10^−3^ M) and K^+^ (100 μL, 1.0 × 10^−3^ M) CH_3_CN respectively. The liner relationship between intensity of UV at 277.9 nm and different concentration of G, K^+^ were carried. The host was 1.0 × 10^−4^ M 2.0 mL CH_3_CN solution and then different amount of **G**, K^+^ were adding into the host solution. The added **G**, K^+^ were 0, 20 40, 60, 80, 100, 120, 140, 160, 180, 200 μL, respectively.

### DFT computational details

The calculations reported in this article were performed using Gaussian03 program package. The structure of **C4C5** and the complex of **C4C5**, **G** and K^+^ have been optimized by going through a cascade process starting from HF/3-21G → HF/6-31G → B_3_LYP/3-21G → B_3_LYP/6-31G. The frequency analysis calculations were performed and the absence of imaginary frequencies indicated the low energy minimum of the structures obtained.

### Preparation of the Si-N_3_-modified silicon substrates

#### Fabrication of the micro–Si interface

A silicon wafer was used directly as the smooth substrate. The structured silicon substrate was fabricated by the combination of photolithography and inductively coupled plasma (ICP) deep etching techniques. The photolithography and ICP techniques were used to obtain the patterned silicon micropillar structure on the silicon wafer. A rough surface introduced geometrical structures with patterned square pillars on a flat silicon wafer, 20 μm high, 9 μm long and with a spacing of 12 μm between the silicon pillars^51^.

#### Preparation of the Si-N_3_ -modified silicon substrates

Silicon substrates cut into 1 × 1 cm^2^ square pieces were soaked in chromosulfuric acid solution for 30 minutes and then rinsed with double distilled water and dried under a stream of N_2_ gas. Cleaned wafers were immersed in aqueous NaOH (0.1 mol·L^−1^) for 6 minutes and subsequently in HNO_3_ (0.1 mol·L^−1^) for 12 minutes to generate surface hydroxyl groups. Then the silicon substrates had been washed by an excess of double distilled water and dried under N_2_ flow. After the silicon substrates had been washed with an excess of water and dried under a stream of N_2_ flow, they were immersed in a refluxed solution of 5 wt% Si-N_3_ in dry toluene (10 mL) at 110°C for 6 h. Then they were washed with toluene and chloroform to remove excess Si-N_3_ and dried under a stream of N_2_ gas.

#### The click reaction between Si-N_3_ and C4C5 on silicon substrates

The silicon surfaces modified Si-N_3_ were immersed in **C4C5** solution in CH_3_CN at 10^−3^ M, then the mixture of copper sulfate(10^−6^ M) and sodium ascorbate (10^−7^ M) were added into this solution, and heated the solution at 75°C for 8 hours. Then the silicon wafer were washed with little CH_3_CN and dried under a stream of N_2_ gas.

## Author Contributions

N.M.F., D.M.T., H.T.D. and H.B.L. designed research; L.L. and N.M.F. performed research; N.M.F. synthesized compounds; L.L. and N.M.F. analysed data; L.L. done the calculation; L.L., X.Y.Z. and H.B.L. wrote the paper.

## Supplementary Material

Supplementary Information Cation-Induced Pesticide Binding and Release by a Functionalized Calix[4]arene Molecular Host
