# Supplementary material for: Cation-Induced Pesticide Binding and Release by a Functionalized Calix[4]arene Molecular Host
Source: Sci Rep. 2015 Mar 11;5:8982. doi: 10.1038/srep08982 (PMC4355745; doi:10.1038/srep08982)
Supplement: Supplementary Information — Cation-Induced Pesticide Binding and Release by a Functionalized Calix[4]arene Molecular Host [file srep08982-s1.docx]

Supporting Information for

Cation-Induced Pesticide Binding and Release by a Functionalized Calix[4]arene Molecular Host

Li Luo, Xiaoyan Zhang , Ningmei Feng , Demei Tian ,Hongtao Deng and Haibing Li*

Key Laboratory of Pesticide & Chemical Biology (CCNU), Ministry of Education; College of Chemistry, Central China Normal University, Wuhan 430079, PR China [lhbing@mail.ccnu.edu.cn](mailto:lhbing@mail.ccnu.edu.cn).

1. ^1^H NMR, ^13^C NMR and ESI-MS spectrum of **C4C5** **S2-S4**

2. UV spectrum and Job plot **S5-S6**

3. The association constant of **C4C5** and **G**, **C4C5** and K^+^ **S7-S8**

3. ^1^H NMR and ESI-MS of K^+^-controlled binding/ release **G** **S9-S10**

4. Gaussian calculation **S11-S19**

5. K^+^-controlled binding/ release **G** on the surface **S20-S22**

**^1^H NMR spectrum of C4C5**

**Fig. S1.** ^1^H NMR spectrum of **C4C5** (CD_3_CN, 400MHz).

**13C NMR spectrum of C4C5**


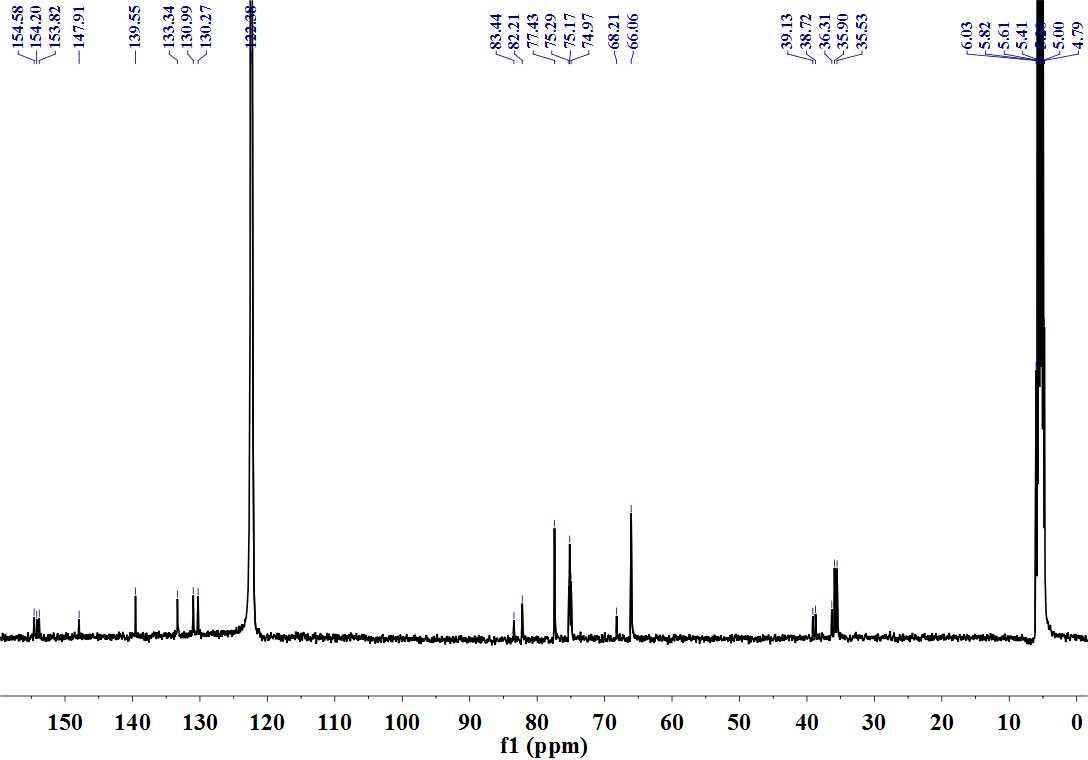


**Fig. S2.** ^13^C NMR spectrum of **C4C5** (CD_3_CN,400MHz).

**ESI-MS of C4C5**


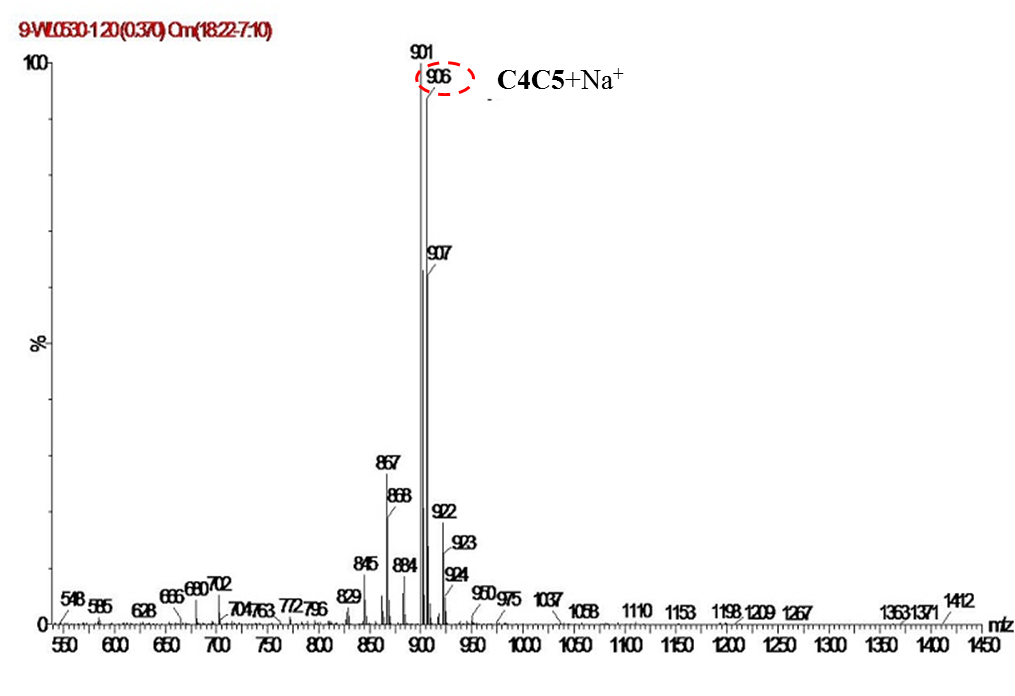


**Fig. S3.** ESI-MS spectrum of **C4C5**.

**Ultraviolet-visible (UV) spectrums**


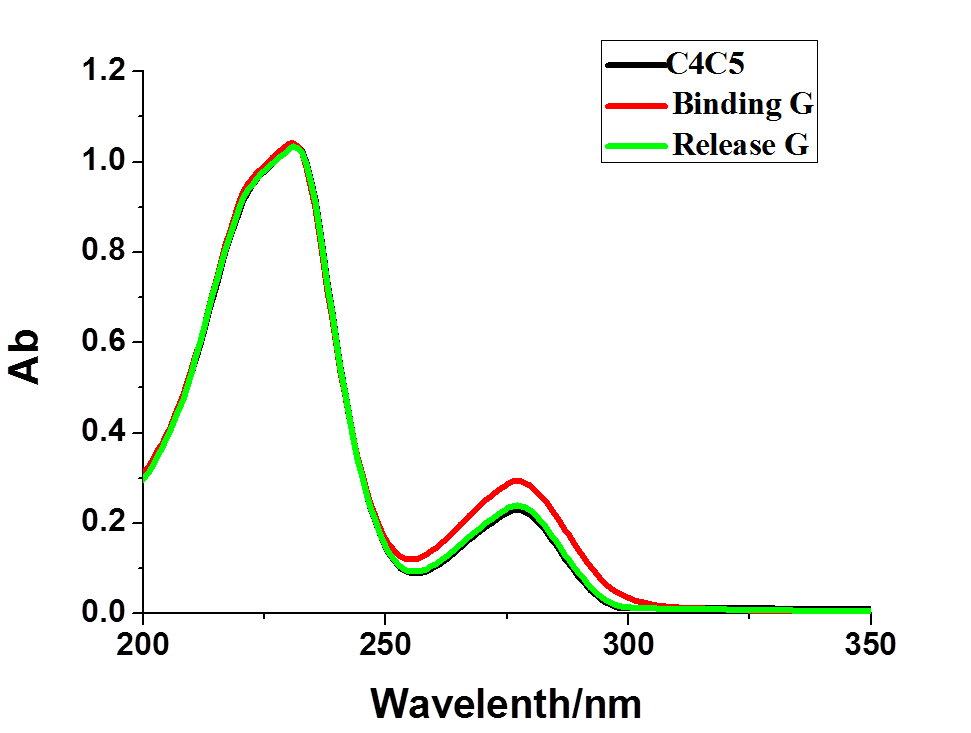


**Fig. S4.** UV changes of **C4C5**, binding **G** and release **G** in CH_3_CN (black line represents the UV spectrum of **C4C5**; red line represents binding **G**; green line represents release **G**).

**The Job plot of C4C5 +G, C4 5+K^+^ and association constants of 4C5 +G, C4 5+K^+^**


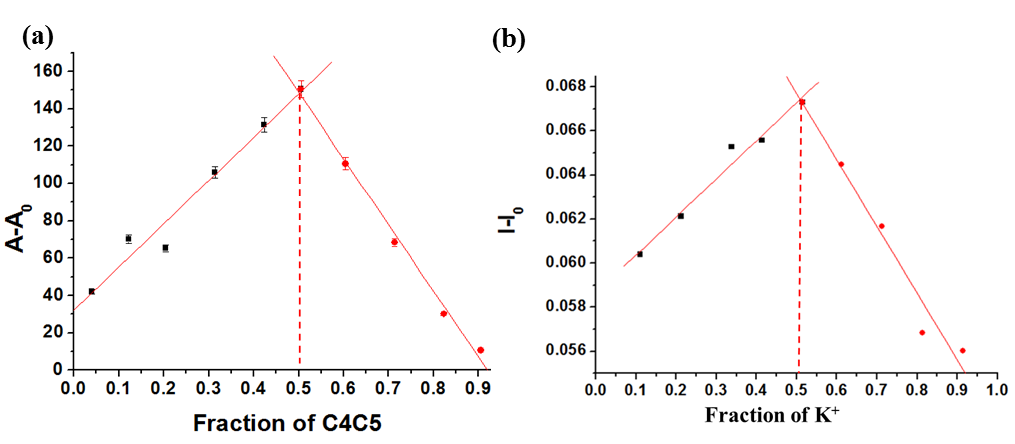


**Fig. S5. (a)** Continuous variation plot of the **C4C5** and **G** system. Job plot indicates the conjugating ratio of **C4C5** and **G** was 1:1. (b) Continuous variation plot of the **C4C5** and K^+^ system. Job plot indicates the conjugating ratio of **C4C5** and K^+^ was 1:1


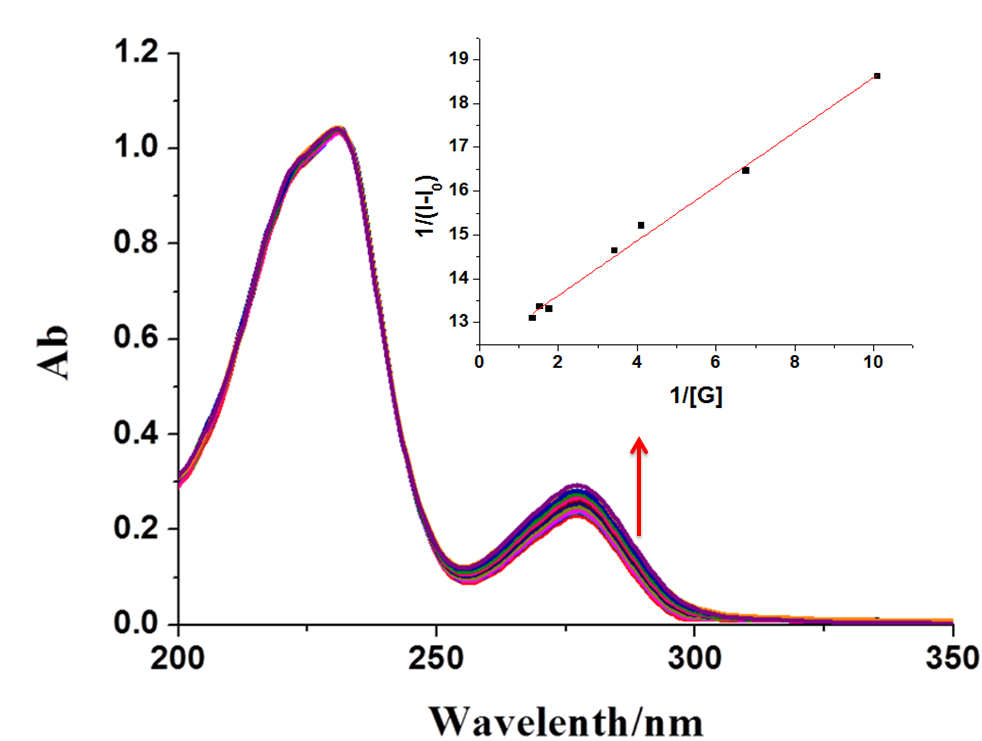


**Fig. S6.** The relationship between 277.9 nm intensity and different concentration of **G**: 1.96 ×10^-5^, 2.90×10^-5^ , 4.7 ×10^-5^, 5.66×10^-5^, 6.54×10^-5^, 7.40×10^-5^, 9.09×10^-5^. The insert shows the linear relationship with the increasing concentration of **G**. The association canstant was 6.22×10^3^ M^-1^


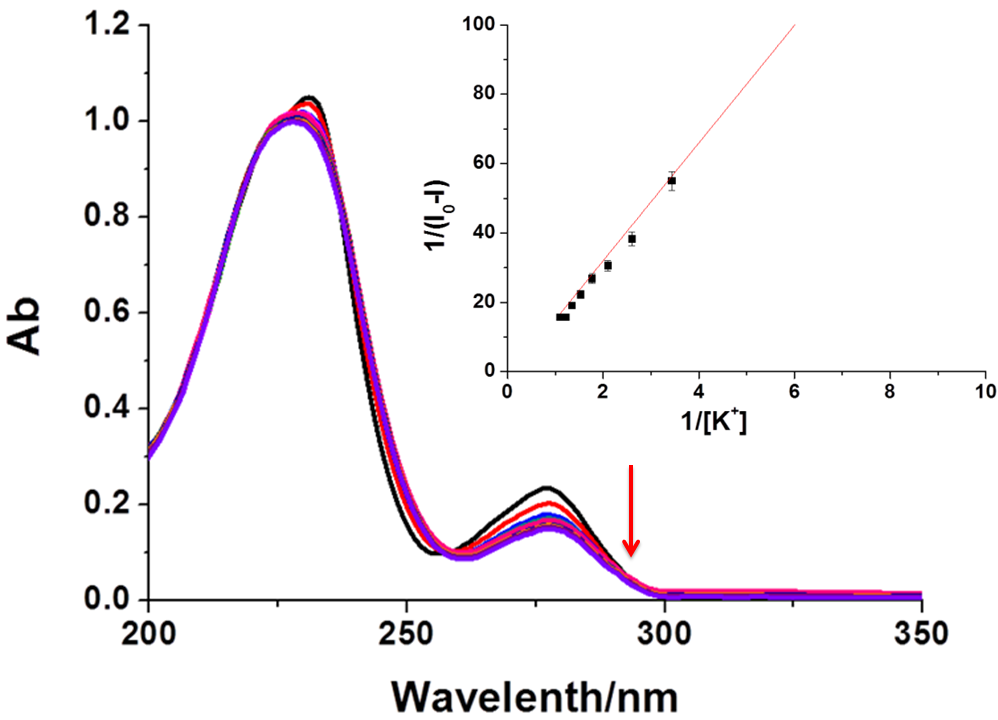


**Fig. S7.** The relationship between 277.9 nm intensity and different concentration of K^+^: 1.96 ×10^-5^, 2.90×10^-5^ , 4.7 ×10^-5^, 5.66×10^-5^, 6.54×10^-5^, 7.40×10^-5^, 9.09×10^-5^. The insert shows the linear relationship with the increasing concentration of K^+^. The association constant was 1.04×10^5^ M^-1^.

**^1^H NMR spectrum of K^+^-controlled binding/release G**

**Fig. S8.** ^1^H NMR spectra (400 MHz, CD_3_CN, 295K) of (a) free **C4C5**; (b) free carbaryl; (c) carbaryl and 1.0 equiv of **C4C5**, (d) to the solution of (c) was added 1.0 equiv of KClO_4_;(e) to the solution of (d) was added 1.0 equiv of **18C6**.

**ESI-MS**


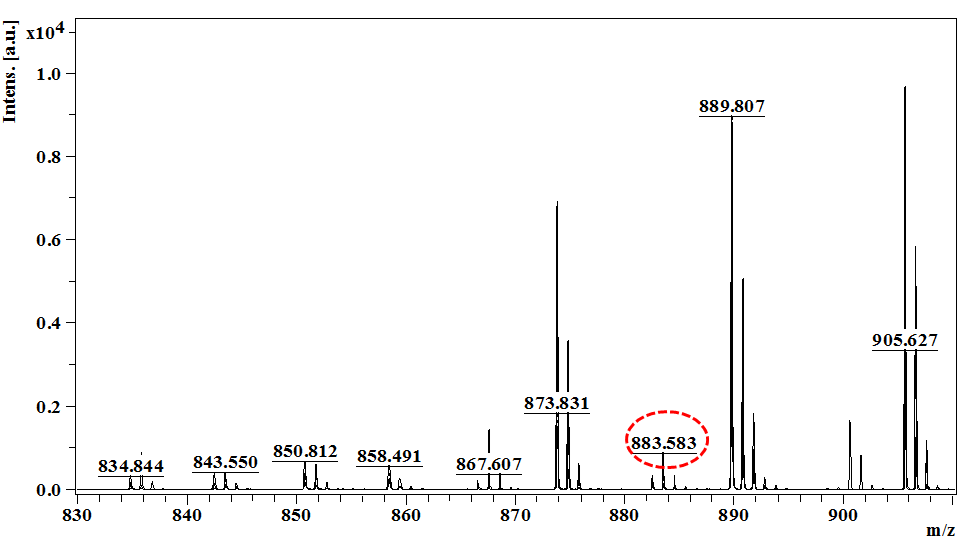


**Fig. S9. 18C6** was added into [**C4C5**+K^+^] complex, m/z= 883.58 appeared in the ESI mass spectrum can be attributed to [**C4C5**+H], which can illustrate K^+^ release from **C4C5**.

**Gaussian calculation of C4C5, C4C5 and G, C4C5 and K^+^**

| B3LYP/6-31G (**C4C5**) | -2780.1868a.u. |
| --- | --- |
| B3LYP/6-31G（**C4C5** and **G**） | -3449.3115a.u. |
| B3LYP/6-31G (**G**) | -669.12334a.u. |
| B3LYP/6-31G (**C4C5** + K^+^) | -3380.2316a.u. |
| ΔE=E**_C4C5-G_ -** (E**_C4C5_ +** E**_G_**) | -0.0014a.u. |
| ΔE_2_= E**_C4C5-K+_ -** (E**_C4C5_** + E**_K+_**) | -0.9438 a.u. |

**Fig. S10.** The table demonstrated energy of **C4C5**, **C4C5** and **G**, **C4C5** and K^+^ at Gaussian calculation based on the equation of ΔE =E _(host-guest)_-(E _host_ + E _guest_), the binding energy of **C4C5** and K^+^ was much bigger than **C4C5** and **G**, which indicated that [**C4C5**+K^+^] complex is much stable than [**C4C5+G]** complex.

| O | -2.63422500 | -1.26694700 | -0.06166600 |
| --- | --- | --- | --- |
| O | -2.24490700 | 1.87651000 | -0.29652100 |
| C | 2.70071100 | 5.95453000 | -1.63086500 |
| C | 2.91613400 | 5.65953800 | 0.84987900 |
| C | 1.17772100 | 7.23355500 | -0.09388400 |
| C | 1.93435200 | -5.82265300 | 0.22210100 |
| C | -0.20108500 | -7.16195400 | 0.43582400 |
| C | -1.94713800 | 0.59195900 | 2.63689100 |
| O | -2.62021400 | 0.07189000 | -2.83410700 |
| C | 0.87693400 | -6.15648900 | 2.46959400 |
| C | 0.57749600 | -5.92730300 | 0.97012700 |
| C | 0.84743500 | 4.74729600 | -0.32755800 |
| C | 0.49095200 | 4.06578100 | -1.50334300 |
| C | -0.53679200 | 3.10561700 | -1.53527800 |
| C | -1.14086300 | 1.74263200 | 2.74432300 |
| C | 0.09969700 | 1.62580500 | 3.38535400 |
| C | 0.57196400 | 0.40461000 | 3.89877000 |
| C | -0.19719400 | -0.73869900 | 3.63122600 |
| C | -1.44731700 | -0.67306400 | 2.99148500 |
| C | 1.53361300 | -0.60390000 | -3.21462900 |
| C | 0.65405600 | -1.66291600 | -2.92769000 |
| C | -0.71285300 | -1.46603800 | -2.69392600 |
| C | -0.86506300 | 2.38306600 | -2.84308200 |
| C | 1.82897900 | 0.37209100 | 4.79791400 |
| C | 1.90432500 | 5.87524600 | -0.30751800 |
| C | 2.97379000 | -0.90457800 | -3.68901200 |
| C | 1.01472300 | 0.69767600 | -3.13211500 |
| C | -1.22882900 | 2.83990000 | -0.34179500 |
| C | -0.53618900 | -4.23833800 | -0.59086500 |
| O | -3.29325300 | 0.73391800 | 2.21105000 |
| C | 1.45672100 | 1.01770900 | 6.16434800 |
| C | 3.68475500 | -1.89768900 | -2.73369000 |
| C | 2.99837200 | 1.17356700 | 4.16994600 |
| C | 3.83711600 | 0.37416400 | -3.79014300 |
| C | 2.32460000 | -1.06889900 | 5.06337700 |
| C | 2.89104000 | -1.54363300 | -5.10561600 |
| C | -1.21860300 | -0.15507900 | -2.79471900 |
| C | -0.35274400 | 0.94534500 | -2.91810100 |
| C | -1.58724700 | -2.65724400 | -2.30201900 |
| C | -1.33083800 | -3.12084400 | -0.86987500 |
| C | -1.57760100 | 3.09062800 | 2.17515500 |
| C | 0.15841600 | 4.40870000 | 0.85288600 |
| C | -0.86867900 | 3.45771200 | 0.87214800 |
| C | -0.26495100 | -4.65441000 | 0.72754400 |
| C | -2.21233900 | -1.96260400 | 2.69469600 |
| C | -1.88362800 | -2.40724200 | 0.20944700 |
| C | -0.81407000 | -3.89617800 | 1.77591000 |
| C | -1.62381100 | -2.77143200 | 1.53976500 |
| C | -3.61208000 | 2.23231600 | -0.72065700 |
| C | -4.68472300 | -2.68168700 | -0.15649500 |
| C | -3.21034600 | -0.21523600 | -4.14811700 |
| C | -4.24435800 | 0.86875400 | 3.32060300 |
| C | -4.53499000 | 4.58013000 | 0.17660700 |
| C | -5.57746500 | 1.31357600 | 2.76538200 |
| O | -6.12906300 | 0.24044700 | 1.95724000 |
| C | -7.46233300 | 0.51112000 | 1.45004200 |
| C | -4.64646500 | 0.25377300 | -4.15662500 |
| O | -5.41826100 | -0.57582500 | -3.25457800 |
| C | -6.83591500 | -0.25938600 | -3.22913300 |
| C | -7.78884800 | -0.50023200 | 0.37233000 |
| O | -7.14264700 | -0.07252500 | -0.85745400 |
| C | -7.45767400 | -0.90187800 | -2.00842300 |
| C | -4.11914700 | -1.33734800 | -0.20735700 |
| C | -4.10693200 | 3.51407400 | -0.21704300 |
| C | -5.16909200 | -3.79519700 | -0.11323100 |
| H | 2.05482700 | 6.20069700 | -2.48107000 |
| H | 3.21189100 | 5.00959600 | -1.85063900 |
| H | 3.46094600 | 6.74046100 | -1.55619900 |
| H | 2.42720900 | 5.67612800 | 1.82909300 |
| H | 3.43374500 | 4.69935100 | 0.75049800 |
| H | 3.66812900 | 6.45807500 | 0.84291000 |
| H | 0.62160800 | 7.23979300 | 0.84991500 |
| H | 0.46669500 | 7.42534200 | -0.90568700 |
| H | 1.90352300 | 8.05637500 | -0.06884100 |
| H | 2.50782800 | -4.95640300 | 0.57049000 |
| H | 1.79634800 | -5.71909600 | -0.85912900 |
| H | 2.53316000 | -6.72493900 | 0.39777000 |
| H | -1.15616900 | -7.27496700 | 0.96158100 |
| H | -0.41531500 | -7.06548900 | -0.63405100 |
| H | 0.38533600 | -8.07754100 | 0.58481700 |
| H | -0.04092200 | -6.30660600 | 3.04908500 |
| H | 1.42479300 | -5.31235600 | 2.90462600 |
| H | 1.49439200 | -7.05434500 | 2.58806200 |
| H | 1.00345800 | 4.28498500 | -2.43318800 |
| H | 0.69407200 | 2.52609200 | 3.50674500 |
| H | 0.15745400 | -1.71461700 | 3.94221600 |
| H | 1.02960800 | -2.68097700 | -2.89857000 |
| H | -0.42065200 | 2.96117200 | -3.66278900 |
| H | -1.94448800 | 2.37477200 | -3.00544000 |
| H | 1.66712100 | 1.55267400 | -3.26902600 |
| H | -0.13135600 | -4.80271700 | -1.42623900 |
| H | 0.64250400 | 0.46496600 | 6.64760600 |
| H | 2.32374100 | 1.01286800 | 6.83726800 |
| H | 1.13051200 | 2.05576900 | 6.03438100 |
| H | 3.15972700 | -2.85650500 | -2.67383000 |
| H | 3.75328500 | -1.49031600 | -1.72196100 |
| H | 4.70018500 | -2.10289700 | -3.09736400 |
| H | 3.29160100 | 0.74953400 | 3.20589600 |
| H | 2.73904100 | 2.22607100 | 4.01445300 |
| H | 3.86847200 | 1.14434100 | 4.83703200 |
| H | 3.91955700 | 0.88512800 | -2.82488800 |
| H | 3.43067100 | 1.08102700 | -4.52260600 |
| H | 4.84818000 | 0.10670500 | -4.12133900 |
| H | 2.57840800 | -1.58293100 | 4.13009500 |
| H | 3.22698600 | -1.03692700 | 5.68475300 |
| H | 1.57717800 | -1.66519300 | 5.59923300 |
| H | 2.31347700 | -2.47434700 | -5.08697300 |
| H | 3.89691000 | -1.77317600 | -5.48019600 |
| H | 2.40833400 | -0.85968900 | -5.81350200 |
| H | -2.64074500 | -2.39358300 | -2.41168000 |
| H | -1.38239400 | -3.49531400 | -2.97994200 |
| H | -2.65473900 | 3.07580600 | 2.00713200 |
| H | -1.36506300 | 3.87722600 | 2.90979400 |
| H | 0.40946900 | 4.90716600 | 1.78430500 |
| H | -2.20942400 | -2.59522400 | 3.59120000 |
| H | -3.25264000 | -1.71962800 | 2.46895600 |
| H | -0.63379700 | -4.18415600 | 2.80549100 |
| H | -3.66152500 | 2.20956000 | -1.81380900 |
| H | -4.21387300 | 1.40741100 | -0.34135300 |
| H | -3.16483600 | -1.29073300 | -4.35351400 |
| H | -2.64595400 | 0.31518100 | -4.92744100 |
| H | -3.87663900 | 1.61895600 | 4.03387500 |
| H | -4.34999900 | -0.08991100 | 3.84301100 |
| H | -4.89851800 | 5.52194700 | 0.51362900 |
| H | -5.45586000 | 2.22038000 | 2.15757300 |
| H | -6.25487000 | 1.54044400 | 3.60513600 |
| H | -8.19216400 | 0.43757800 | 2.27182600 |
| H | -7.51660500 | 1.52201900 | 1.02280600 |
| H | -5.03981100 | 0.17074200 | -5.18325000 |
| H | -4.70819000 | 1.30766400 | -3.84938000 |
| H | -7.31571200 | -0.63821200 | -4.14512700 |
| H | -6.98777600 | 0.82784700 | -3.17733700 |
| H | -7.43623600 | -1.49619500 | 0.67042800 |
| H | -8.88032000 | -0.54054900 | 0.22232800 |
| H | -8.55106000 | -0.95697400 | -2.13886900 |
| H | -7.06515200 | -1.91790000 | -1.87365200 |
| H | -4.33343500 | -0.87087600 | -1.17114400 |
| H | -4.52957500 | -0.71701700 | 0.59242400 |
| H | -5.57341300 | -4.77851700 | -0.06972800 |
| C | 3.91052100 | -2.22298200 | 1.36374800 |
| C | 5.12419800 | -1.59706600 | 1.15323400 |
| C | 5.16426400 | -0.26405900 | 0.66056400 |
| C | 3.93159200 | 0.43180100 | 0.40121100 |
| C | 2.70102700 | -0.24994000 | 0.62600800 |
| C | 2.68973800 | -1.55047500 | 1.09179100 |
| C | 6.38457500 | 0.42822600 | 0.40256200 |
| C | 6.40559500 | 1.72942700 | -0.05485500 |
| C | 5.18288000 | 2.40719900 | -0.29663000 |
| C | 3.97478400 | 1.77394500 | -0.07767400 |
| O | 7.60238900 | -0.23500000 | 0.69258600 |
| C | 8.37224000 | -0.87958800 | -0.30734100 |
| N | 7.87368000 | -0.79657600 | -1.57439100 |
| C | 8.55458500 | -1.42690500 | -2.70306000 |
| O | 9.40828500 | -1.45664900 | 0.02945100 |
| H | 3.88792500 | -3.23975100 | 1.74310900 |
| H | 6.05796500 | -2.10119000 | 1.37257200 |
| H | 1.76923900 | 0.26848700 | 0.42591600 |
| H | 1.74506700 | -2.05786600 | 1.25372300 |
| H | 7.35717900 | 2.22454300 | -0.21206500 |
| H | 5.20769400 | 3.43106700 | -0.65428500 |
| H | 3.03787000 | 2.28945800 | -0.26550300 |
| H | 7.01471100 | -0.29345500 | -1.74037100 |
| H | 7.90887800 | -2.17010800 | -3.18367100 |
| H | 8.85525500 | -0.68123900 | -3.44754100 |
| H | 9.44630000 | -1.92531400 | -2.32043600 |

**Fig. S11.** Cartesian coordinates for B_3_LYP/6-31G optimized structure of **C4C5** and **G**.

| O | -1.20715100 | -2.76689800 | 0.05779800 |
| --- | --- | --- | --- |
| O | -2.04878600 | 1.88462400 | -0.29575700 |
| C | 2.71741000 | 6.04337500 | 1.31442600 |
| C | 1.90412600 | 6.78084700 | -0.94308400 |
| C | 3.68124600 | 4.98415700 | -0.74691900 |
| C | 4.74611800 | -4.78725100 | 1.80939500 |
| C | 5.26117800 | -3.67947300 | -0.38157300 |
| C | -0.09204500 | -0.26983100 | -2.73850500 |
| O | -1.76325000 | -0.39579500 | 2.44902600 |
| C | 4.21153500 | -5.98503800 | -0.33169600 |
| C | 4.24718600 | -4.59331100 | 0.35925900 |
| C | 1.27956900 | 4.47629600 | -0.12126100 |
| C | 0.68041000 | 3.99426600 | 1.05156500 |
| C | -0.37110100 | 3.05952200 | 1.03337500 |
| C | 0.36685100 | 1.04659100 | -2.92595500 |
| C | 1.71906700 | 1.23553100 | -3.25435500 |
| C | 2.62123100 | 0.17106200 | -3.37233700 |
| C | 2.13366800 | -1.11747100 | -3.09608200 |
| C | 0.79416800 | -1.36656200 | -2.77076500 |
| C | 2.25840500 | 0.48591500 | 3.45103600 |
| C | 1.81582600 | -0.83436200 | 3.29992200 |
| C | 0.50125600 | -1.14653600 | 2.91763700 |
| C | -0.87626400 | 2.42961800 | 2.33097500 |
| C | 4.10075200 | 0.36928200 | -3.76622700 |
| C | 2.38309900 | 5.55663800 | -0.11389700 |
| C | 3.69227700 | 0.84116300 | 3.89968100 |
| C | 1.34571200 | 1.51060400 | 3.14798300 |
| C | -0.85368400 | 2.65358800 | -0.21810500 |
| C | 2.11511900 | -3.62437300 | 1.45746600 |
| O | -1.48317100 | -0.52211800 | -2.53697100 |
| C | 4.43206900 | 1.84696000 | -4.07776000 |
| C | 4.53817400 | -0.41605800 | 4.20599600 |
| C | 4.41709600 | -0.47328000 | -5.03280300 |
| C | 4.40269600 | 1.64011400 | 2.77175600 |
| C | 5.01163200 | -0.09642200 | -2.59717200 |
| C | 3.63285700 | 1.71038300 | 5.18606900 |
| C | -0.39582300 | -0.08555200 | 2.70403600 |
| C | 0.02469500 | 1.25938400 | 2.75550200 |
| C | 0.11803600 | -2.61073000 | 2.66762700 |
| C | 0.80100800 | -3.12860700 | 1.40162900 |
| C | -0.52118400 | 2.28113000 | -2.72888700 |
| C | 0.85130400 | 3.92146400 | -1.34233800 |
| C | -0.19284500 | 2.99205200 | -1.41441600 |
| C | 2.83699300 | -3.96589600 | 0.30409800 |
| C | 0.35774900 | -2.79225400 | -2.40758200 |
| C | 0.19806600 | -3.01995200 | 0.13931000 |
| C | 2.23130400 | -3.70505600 | -0.93933800 |
| C | 0.92412400 | -3.21163400 | -1.05039300 |
| C | -1.96418000 | -4.05035400 | 0.04947100 |
| C | -3.23436900 | 2.65121000 | -0.76874700 |
| C | -3.38669500 | -3.76845200 | -0.06486600 |
| C | -4.57074000 | -3.51922100 | -0.15763300 |
| C | -2.62043200 | -0.28226700 | 3.64145100 |
| C | -2.25790800 | -0.52524000 | -3.78864300 |
| C | -4.66073700 | 3.68490900 | 1.24081200 |
| C | -3.60407500 | -1.16433200 | -3.54962600 |
| O | -4.36907300 | -0.35776600 | -2.60041700 |
| C | -5.79342500 | -0.67789600 | -2.60949200 |
| C | -3.91153700 | -1.02621200 | 3.39486700 |
| O | -4.59922900 | -0.39819900 | 2.27559000 |
| C | -6.01037400 | -0.73627800 | 2.17461600 |
| C | -6.46088500 | 0.03263500 | -1.45635500 |
| O | -5.98793500 | -0.57650500 | -0.22164500 |
| C | -6.57825400 | -0.00163600 | 0.98336400 |
| H | 3.10735200 | 5.23165200 | 1.93999100 |
| H | 1.84140900 | 6.47566100 | 1.81119400 |
| H | 3.48792000 | 6.82016400 | 1.26490300 |
| H | 1.68709900 | 6.50668100 | -1.98107000 |
| H | 0.99507900 | 7.21394700 | -0.51040100 |
| H | 2.67982900 | 7.55547900 | -0.95504800 |
| H | 3.52246400 | 4.66980800 | -1.78415000 |
| H | 4.04199300 | 4.11804300 | -0.18029600 |
| H | 4.46863900 | 5.74695400 | -0.74602100 |
| H | 4.08644800 | -5.44940400 | 2.38168000 |
| H | 4.82893500 | -3.83258100 | 2.34215400 |
| H | 5.74123100 | -5.24441100 | 1.79436900 |
| H | 4.99837300 | -3.55333200 | -1.43726800 |
| H | 5.30286400 | -2.68628500 | 0.07983100 |
| H | 6.26433600 | -4.11951400 | -0.33913500 |
| H | 3.90028800 | -5.90882000 | -1.37908400 |
| H | 3.51433500 | -6.65755000 | 0.18091800 |
| H | 5.20740600 | -6.44291100 | -0.30886600 |
| H | 1.01602200 | 4.35899100 | 2.01405000 |
| H | 2.06183700 | 2.24859700 | -3.41905000 |
| H | 2.81466900 | -1.95999800 | -3.13696100 |
| H | 2.50050500 | -1.65289700 | 3.47781700 |
| H | -0.87983300 | 3.18840500 | 3.12283000 |
| H | -1.90057600 | 2.08430900 | 2.19409200 |
| H | 1.67256700 | 2.54196600 | 3.21058800 |
| H | 2.56702800 | -3.75362500 | 2.43292700 |
| H | 3.82579600 | 2.23313300 | -4.90542200 |
| H | 4.28026700 | 2.49118400 | -3.20383100 |
| H | 5.48424000 | 1.93125400 | -4.37031100 |
| H | 4.09413900 | -1.01749600 | 5.00757100 |
| H | 4.65732200 | -1.05019100 | 3.31952500 |
| H | 5.53881100 | -0.11372000 | 4.53320800 |
| H | 4.24452800 | -1.54172900 | -4.86502600 |
| H | 3.79390500 | -0.15793500 | -5.87740500 |
| H | 5.46804300 | -0.34482400 | -5.31688800 |
| H | 4.46369600 | 1.04572100 | 1.85280400 |
| H | 5.42151200 | 1.90293000 | 3.08011100 |
| H | 3.87151100 | 2.56896700 | 2.53733700 |
| H | 4.85514600 | -1.15345500 | -2.35857500 |
| H | 4.81101500 | 0.48739300 | -1.69142700 |
| H | 6.06652200 | 0.03646900 | -2.86495600 |
| H | 3.14636300 | 1.16575900 | 6.00327500 |
| H | 4.64659000 | 1.97625600 | 5.50797900 |
| H | 3.07861300 | 2.64066700 | 5.02226700 |
| H | 0.43529000 | -3.21994400 | 3.52213900 |
| H | -0.96577900 | -2.69133600 | 2.57818300 |
| H | -0.35629700 | 2.97426700 | -3.56214600 |
| H | -1.56920500 | 1.98460600 | -2.74783000 |
| H | 1.33726000 | 4.22202500 | -2.26490000 |
| H | -0.73137500 | -2.84117900 | -2.39156700 |
| H | 0.71856000 | -3.48486200 | -3.17721600 |
| H | 2.78321100 | -3.90451700 | -1.85193800 |
| H | -1.74025000 | -4.59449200 | 0.97537000 |
| H | -1.61103800 | -4.65963500 | -0.79164300 |
| C | -4.01181300 | 3.21517300 | 0.32939400 |
| H | -3.81781400 | 1.93251900 | -1.35358900 |
| H | -5.59860500 | -3.25016900 | -0.23225300 |
| H | -2.82249000 | 0.77505500 | 3.84357900 |
| H | -2.10038900 | -0.71911700 | 4.50242400 |
| H | -1.71347100 | -1.09937300 | -4.54853300 |
| H | -2.38251300 | 0.50234000 | -4.14752600 |
| H | -4.13944600 | -1.21678100 | -4.50878700 |
| H | -3.49309700 | -2.18253900 | -3.15303100 |
| H | -5.93350200 | -1.76227500 | -2.50882500 |
| H | -6.23877900 | -0.35279100 | -3.56036400 |
| H | -4.53223000 | -0.96701700 | 4.30115600 |
| H | -3.72084500 | -2.08410300 | 3.16806800 |
| H | -6.53638700 | -0.42342800 | 3.08836000 |
| H | -6.13630600 | -1.82069200 | 2.05171400 |
| H | -7.55233200 | -0.07704100 | -1.53851300 |
| H | -6.21841700 | 1.10413200 | -1.46846900 |
| H | -7.67139300 | -0.11902600 | 0.95748400 |
| H | -6.33176600 | 1.06616900 | 1.04630000 |
| H | -5.21676200 | 4.12587200 | 2.03540400 |
| H | -2.89182700 | 3.44305900 | -1.44430600 |
| K | -2.88280700 | -0.60379900 | -0.04502100 |

**Fig. S12.** Cartesian coordinates for B_3_LYP/6-31G optimized structure of **C4C5** and K^+^.

**SEM image of silicon before and after C4C5 modified**


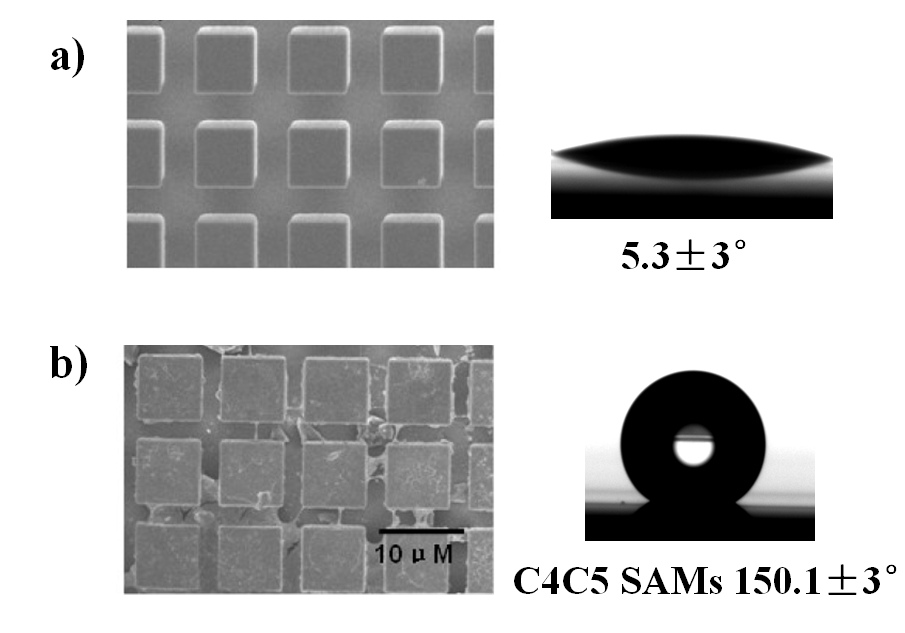


**Fig. S13**. (a)SEM image of the rough silicon surface before **C4C5** modified and water-drop profiles on bare silicon. (b) SEM image of rough silicon after **C4C5** modified and water-drop profile **C4C5** SAMs, indicating that **C4C5** has successfully modified on the silicon.

**XPS of Si-N_3_ and C4C5 on the surface**

**Fig. S14.** (a) Water contact angle of Si-N_3_ and X-ray photoelectron spectra of Si-N_3_ modified silicon substrate. (b) Water contact angle of **C4C5** and X-ray photoelectron spectra of **C4C5** modified silicon surface.The XPS and CA have demonstrated **C4C5** modified on the silicon surface.

**K^+^-controlled binding/release G on the surface**

**Fig. S15.** CA relationship images for **C4C5** SAMs with **G**, K^+^, **18C6**.
